# Selective cold pain inhibition by targeted block of TRPM8-expressing neurons with quaternary lidocaine derivative QX-314

**DOI:** 10.1038/s42003-018-0062-2

**Published:** 2018-05-31

**Authors:** Serra Ongun, Angela Sarkisian, David D. McKemy

**Affiliations:** 1 Neurobiology Section, Department of Biological Sciences, University of Southern California, Los Angeles, CA 90089 USA; 2 Molecular and Computational Biology Graduate Program, Department of Biological Sciences, University of Southern California, Los Angeles, CA 90089 USA

## Abstract

Treatment of pain with local anesthetics leads to an unfavorable decrease in general sensory acuity due to their indiscriminate block of both pain sensing (nociceptors) and non-pain sensing nerves. However, the cell impermeant lidocaine derivative QX-314 can be selectively targeted to only nociceptors by permeation through ligand-gated cation channels. Here we show that localized injection of QX-314 with agonists for the menthol receptor TRPM8 specifically blocks cold-evoked behaviors in mice, including cold allodynia and hyperalgesia. Remarkably, cooling stimuli also promotes QX-314-mediated inhibition of cold behaviors, and can be used to block cold allodynia, while retaining relatively normal cold sensation. The effects of both agonist and thermally evoked uptake of QX-314 are TRPM8-dependent, results demonstrating an effective approach to treat localized cold pain without altering general somatosensation.

## Abstract (web summary)

Serra Ongun, Angela Sarkisian and David McKemy show that localized co-injection of lidocaine derivative QX-314 and receptor agonists is able to block cold sensitivity in mice in a targeted way, with implications for treating cold pain associated with injury and disease.

## Introduction

There is a great need for analgesics that inhibit pain without leading to negative side effects such as a loss of consciousness, numbness, or paralysis^1^. Pain is often localized, and approaches that provide relief at only the site affected prevent unwanted complications. Hydrophobic local anesthetics, such as lidocaine, indiscriminately cross the plasma membrane of all nerves and bind to intracellular domains of voltage-gated Na^+^ channels, thereby inhibiting channel activity and neuronal excitability^2^. This leads to analgesia due to nociceptor inhibition, but also results in a general block of low-threshold touch receptors, motor neurons, and autonomic nerve fibers, thereby diminishing basic functions and sensations. In addition, the nearly complete block of sensory acuity produced by local anesthetics hinders the ability to detect potentially damaging environmental conditions, a critical defense mechanism against injury. Thus, there is an overwhelming need for novel approaches that can inhibit selective pain modalities locally, without attenuating general sensory perception.

One approach to block only specific subtypes of sensory afferents uses the quaternary lidocaine derivative QX-314, which is permanently charged and alone cannot cross the plasma membrane. A number of nonselective cation channels can permeate positively charged, large molecules (≤600 Da) via their conduction pores, thereby allowing selective intracellular access of specific molecules to only the cells expressing these channels^3–6^. This property has been employed to target QX-314 (~260 Da) to sensory afferents responsible for heat, mechanical, and itch sensations, thereby allowing for pharmacological phenotyping of these chemically accessible neurons in vivo^7–9^. For example, an intradermal or perineural injection of QX-314 with the nociceptor agonist capsaicin produces deficits in heat and mechanical pain, with no impairment of motor or tactile responses^7,10^. In addition, capsaicin also enhances the permeation of other anesthetics such as *N*-methyl amitriptyline, amitriptyline, and bupivacaine^11^, and this technique has recently been applied clinically in a study showing that topical capsaicin mixed with local anesthetics effectively reduced venipuncture pain in humans^12^.

One pain modality in which QX-314-specific nerve block has not been shown is cold pain. Like other somatosensory modalities, cold is a source of serious discomfort and pain after injury and during many chronic pain states, such as hand fractures or whiplash, diabetic neuropathies, multiple sclerosis, complex regional pain syndrome (CRPS), or as a side effect of many chemotherapeutics^13–21^. The principle mediator of cold in sensory afferents is the nonselective cation channel and menthol receptor TRPM8^22^. TRPM8 channels are activated at temperatures below ~25 °C in vitro and TRPM8-null mice (Trpm8^−/−^) fail to distinguish warm from cool and poorly avoid noxious cold^22–27^. Moreover, cold hypersensitivity associated with inflammatory and neuropathic injury is diminished in the absence of this channel^24,27,28^. Two reports suggested that TRPM8 does not permeate large cations in vitro and in vivo^29,30^, whereas we and others have recently shown uptake of large cations in both heterologous cells and sensory neurons expressing TRPM8 channels, as well as found that co-application of TRPM8 agonists with QX-314 blocked Na^+^ currents in these cells^6,31^. Nonetheless, while these latter studies showed that TRPM8 channels permeate large cations, it was unclear if they can be employed for cell-specific targeting of molecules in vivo.

Here we show that a localized co-injection of QX-314 and the TRPM8 agonists’ menthol or WS-12 lead to an essentially complete, yet transient, block of cold sensitivity in mice. Furthermore, we find that cooling itself is a sufficient stimulus for QX-314-mediated attenuation of cold perception, and that both agonist and cooling-induced uptake of QX-314 is blocked by a TRPM8 antagonist. Finally, both TRPM8 agonists and cooling are able to ameliorate cold pain via QX-314 in models of cold allodynia (when stimuli that would normally be innocuous become painful) and hyperalgesia (pain intensity is increased) associated with inflammatory and chemotherapy-induced peripheral neuropathy (CIPN). Intriguingly, we find that titrating the cooling stimulus to moderate temperatures is sufficient in dampening cold pain without altering normal cold sensation, as well as that of other somatosensory modalities. Taken together, our results demonstrate that targeting charged anesthetics to TRPM8 afferents can be used to treat cold and cold pain associated with injury and disease.

## Results

### Co-injection of QX-314 and TRPM8 agonists blocks acute cold-induced behaviors

As we find that large cations permeate TRPM8 channels expressed in heterologous and native cells, and that QX-314 blocks sodium currents in TRPM8-expressing cells stimulated with TRPM8 agonists^31^, we asked if TRPM8 activation in the presence of QX-314 can alter cold sensitivity in vivo. First, to confirm that in vivo thermal responses can be blocked in our hands using TRP channel activation, we examined heat responses in adult wild-type mice after injection of QX-314 and the TRPV1 agonist capsaicin^8^. As reported previously^7^, intraplantar hind paw co-injection of QX-314 (1%) and 1 µg capsaicin (in 20 µl) attenuated hind paw lift latencies to a radiant heat stimulus (Supplementary Figure 1a, *n* = 4 for each). These effects were observed by 30 min postinjection and lasted up to 90 min with no alteration of heat sensitivity when either QX-314 or capsaicin were injected alone (Supplementary Figure 1b).

**Fig. 1:**
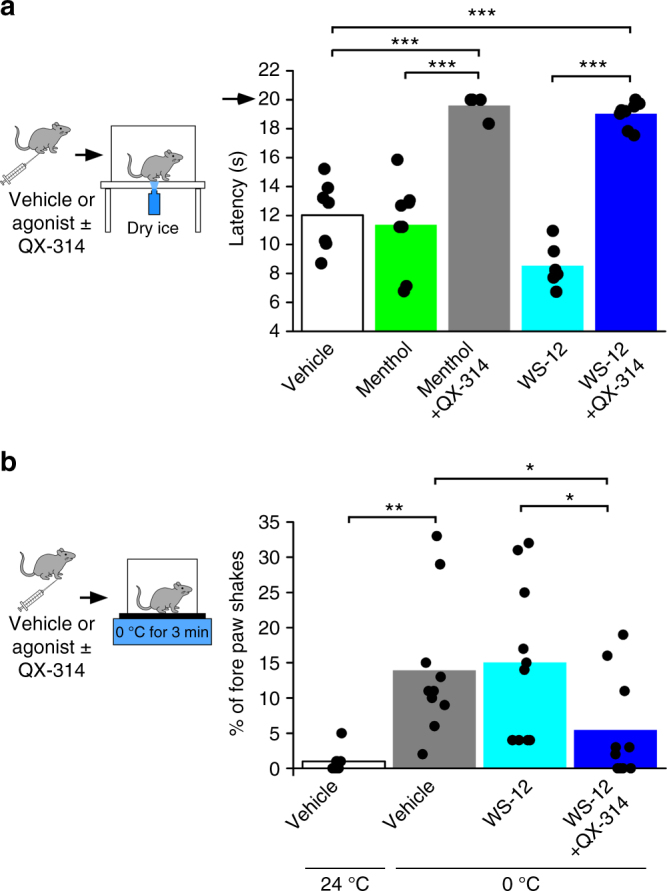
Co-administration of QX-314 with TRPM8 agonists inhibits cold-induced behaviors in vivo. **a** Intraplantar injection of 20 µl of QX-314 (1%) together with menthol (8 µg), resulted in increased in hind paw withdrawal latencies (see model to left) compared to vehicle or menthol alone injections (***p < 0.001 at 90 min postinjection vs. vehicle, *n* = 4–8) in the cold plantar assay. Co-injection of QX-314 (1%) and WS-12 (20 µg) also produced increased hind paw withdrawal latencies (***p < 0.001 vs. WS-12 or vehicle alone at 90 min postinjection, *n* = 6–8). Arrow denotes the cutoff time for the experiment. **b** Nocifensive fore paw shakes are induced when mice are placed on a cold plate for 3 min (see model to left) at 0 °C, but not at 24 °C in vehicle-injected mice (**p < 0.01, 30 min postinjection, *n* = 4–8). These responses were significantly reduced in mice injected with 10 µl of QX-314 together with WS-12 (20 µg) compared with mice injected with vehicle or WS-12 alone (*p < 0.05, 30 min postinjection, *n* = 8)

**Fig. 2:**
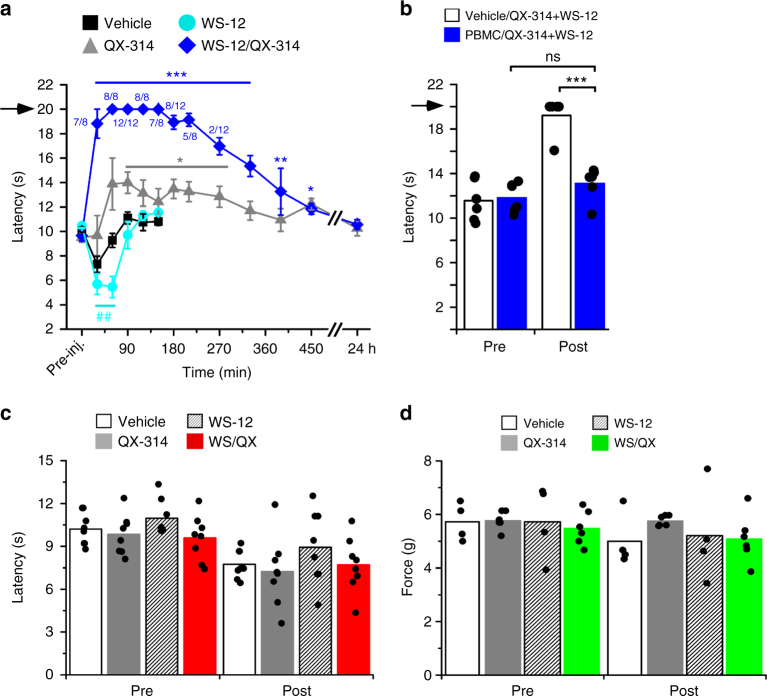
Co-administration of QX-314 and WS-12 produces prolonged and specific inhibition of cold-induced behaviors. **a** Intraplantar injection of QX-314/WS-12 inhibited cold sensitivity up to 5.5 h (***p < 0.001 vs. pre-injection, *n* = 8–12 per time point) with responses remaining inhibited up to 7.5 h postinjection (**p < 0.01, *p < 0.05). WS-12 alone induced cold sensitization during the first 60 min (^##^*p* < 0.05 vs. pre-injection, *n* = 4) and QX-314 alone injected mice displayed inhibitory responses starting at 1 h and lasting up to 4.5 h postinjection (*p < 0.05 vs. pre-injection, *n* = 8–12). Arrow denotes the cutoff time for the experiment. **b** Pre-injection of the TRPM8 antagonist PBMC (100 µg), but not vehicle, prevented QX-314/WS-12 block of cold sensitivity (***p < 0.001, ^NS^*p* > 0.05, *n* = 4–6). **c** Co-injection of QX-314/WS-12 did not alter sensitivity to heat (Hargreaves assay, *n* = 8) or **d** to mechanical stimuli (electronic Von Frey assay, *n* = 4–6; *p* > 0.05 pre- vs. postinjection and between conditions postinjection for both Hargreaves and Von Frey assays)

Next, to determine if TRPM8 agonists, such as menthol, lead to similar effects on cold sensitivity in vivo when co-injected with QX-314, we used the cold plantar assay^32,33^ to measure responses to a radiant cold stimulus. Like capsaicin/QX-314 alterations to heat responses, co-injection of menthol (8 µg) with QX-314 significantly (*p* < 0.01) attenuated lift latencies compared to vehicle or menthol alone (Fig. 1a, *n* = 4–8). Moreover, these effects showed a similar time course as capsaicin/QX-314 injections (Supplementary Figure 2). Menthol is a low-potency TRPM8 agonist and been shown to act nonspecifically at high concentrations, such as those required to elicit a behavioral response^22,34^. However, the TRPM8 agonist WS-12 has a >100-fold higher affinity than menthol and shown to act specifically on TRPM8 in vivo^31,34^. We find that intraplantar injection of 20 µg WS-12 transiently increases cold sensitivity postinjection (Fig. 2a, *n* = 4), with lift latencies returning to basal levels by 90 min. Thus, we repeated the above experiments with WS-12, finding that like menthol, this agonist blocked cold responses when co-injected with QX-314 (Fig. 1a, *n* = 6–8).

The cold plantar assay provides a stimulus that typically induces a hind paw lift when the plate temperature changes by ~1.5–2.0 °C regardless of the basal surface temperature^35^. Thus, it is not a measurement of behaviors evoked by stimuli considered noxious cold. We have shown that fore paw shaking in mice on a 0 °C cold plate is a reliable measure of sensitivity to noxious cold and dependent upon TRPM8 channels and neurons^27^. Specifically, as shown in Supplementary Figure 3, over a 3 min recording period, wild-type mice on a 0 °C plate exhibit robust fore paw shaking compared with when these animals are on the same surface at room temperature (RT) (~23–24 °C, *n* = 4–8), responses that are reduced in Trpm8^−/−^ mice^23^ on the 0 °C surface (*n* = 4). To determine if WS-12 and QX-314 similarly reduce this nocifensive behavior, we injected vehicle, WS-12, or WS-12/QX-314 in both fore paws of wild-type mice and 30 min postinjection we measured the number of fore paw shakes after the animals were placed on the cold plate (Fig. 1b). As with the cold plantar assay, we observed a decrease in the number of shakes in animals injected with WS-12/QX-314 compared with mice injected with vehicle or WS-12 alone (Fig. 1b, *n* = 8). These results demonstrate that QX-314 uptake induced by TRPM8 agonists is able to inhibit both acute innocuous and noxious cold behaviors.

Next, to determine the duration of inhibition of cold, we performed a time course experiment, finding that co-injection of QX-314 with WS-12 essentially blocked cold responses for >3.5 h postinjection (Fig. 2a, *n* = 8–12 per time point). Indeed, many animals failed to respond prior to the cutoff time (20 s) established for the cold plantar assay (fractions above each data point indicate the number of mice that did not respond within the cutoff time)^32^. Cold sensitivity gradually returned but was still attenuated 6–7 h postinjection and did not return to pre-injection values until 24 h later (Fig. 2a).

WS-12 has been shown to be a TRPM8-specific agonist^31,34^, but to confirm that the effects observed are via TRPM8 activation, we tested WS-12/QX-314 block of cold sensation in the presence of the TRPM8 antagonist PBMC^28^. A unilateral intraplantar injection of 100 µg PBMC produces a complete block of cold responses for 4 h postinjection, with behaviors returning to pre-injection levels by 5 h (Supplementary Fig 4a, *n* = 4). As WS-12/QX-314 block of cold behaviors also persists for over 4 h (Fig. 2a), we devised a dual-injection strategy to test PBMC block of WS-12 mediated uptake of QX-314. Wild-type mice were injected with vehicle or PBMC, then again injected as above with QX-314 and WS-12 2 h later and then cold sensitivity was assayed every hour for 3 h post-QX-314/WS-12 injection. At 1 h post-QX-314/WS-12 injection (3 h post PBMC or vehicle injection), cold sensitivity was blocked in both test conditions (Supplementary Figure 4a, *n* = 6), whereas cold sensation returned at 3 h post-QX-314/WS-12 injection (5 h post PBMC or vehicle) in mice that were pre-injected with the TRPM8 antagonist compared to vehicle-injected animals (Fig. 2b). These results demonstrate that TRPM8 antagonism abolishes the ability of WS-12 to induce QX-314 block of cold sensitivity.

Finally, we asked if the WS-12/QX-314 block was specific for cold sensation by measuring responses to heat (Fig. 2c, Hargreaves assay, *n* = 8) and mechanical (Fig. 2d, von Frey, *n* = 4–6) stimuli in mice injected with QX-314 and WS-12 as above, observing no differences in behaviors after the various injections. Thus, these results demonstrate that cold sensitivity can be completely and selectively abolished when QX-314 is coupled with TRPM8 channel activation in vivo.

### Cold stimuli permit QX-314-mediated block of cold behaviors

**Fig. 3:**
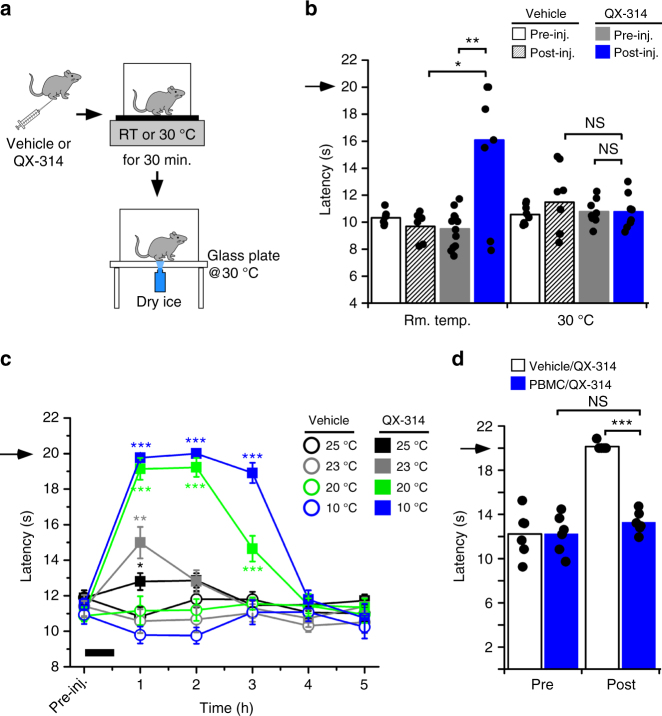
Cold stimuli induced QX-314-mediated inhibition of cold behaviors. **a** Experimental paradigm for **b**, **c**. Upon intraplantar injection of QX-314 or vehicle, mice were placed on a thermally controlled test plate for 30 min and then transferred to the cold plantar apparatus, where the glass surface was warmed to 30 °C, for a 30 min habituation period before testing. **b** Significantly reduced cold responses in QX-314-injected mice exposed to room temperature for 30 min (*p < 0.05 veh. vs. QX-314 at 60 min post-inj.; **p < 0.01 pre- vs. post-QX-314 injection, *n* = 6–12). However, the inhibitory effect of QX-314 alone injection was absent when the QX-314-injected mice were kept at 30 °C after injection (*p* > 0.05 veh. vs. QX-314 or pre- vs. post-QX-314 injection at 60 min post-inj., *n* = 8). **c** A wide range of cold temperatures promote cold specific anesthesia via QX-314. Significant reduction in paw withdrawal latencies was observed up to 3 h when mice were placed on a noxious temperature of 10 °C post-QX-314 injection (***p < 0.001 vs. vehicle or pre-injection) and the innocuous temperature of 20 °C (***p < 0.001 vs. vehicle or pre-inj., *n* = 8). Moreover, milder cold temperatures such as 23 and 25 °C led to significant reductions in cold responsiveness at 1 h after injection (23 °C: **p < 0.01 vs. vehicle or pre-inj.; 25 °C: *p < 0.05 vs. vehicle with *p* = 0.21 vs. pre-inj., *n* = 8 for both temperatures). Bar indicates duration of thermal stimulus and arrows denote the cutoff time for the experiments. **d** Pre-injection of the TRPM8 antagonist PBMC (100 µg), but not vehicle, prevented cooling (10 °C) induced QX-314 block of cold sensitivity (***p < 0.001, ^NS^*p* > 0.05, *n* = 4–6)

In addition to the block observed after co-injection of QX-314 with WS-12, we also noticed a slight increase in lift latencies in mice injected with QX-314 alone (Fig. 2a). This effect was not observed when assessing heat (Fig. 2c) or mechanical stimuli (Fig. 2d) using identical injection parameters, suggesting a specific effect on cold. In the cold plantar assay, upon injection, mice are placed on a glass plate at RT, which varied in our hands from 23 to 24 °C. As these are permissive temperatures for TRPM8 activation in vivo^23,27^, we hypothesized that the reduction in cold sensitivity observed in mice injected with QX-314 alone was due to temperature activation of TRPM8 channels leading to cellular permeation of the Na^+^ channel blocker. To test this, we modified our behavioral paradigm such that immediately after injection mice were placed on a thermally controlled surface (Hot/cold plate, IITC) then transferred to the cold plantar apparatus in which the glass plate was heated to 30 °C, the preferred temperature for mice and nonpermissive for TRPM8 activation^25,27^. To determine if temperature mediated the effects of QX-314 alone, wild-type mice were injected with either vehicle or QX-314 and placed for 30 min on the test plate set at either RT or heated to 30 °C, then transferred to the heated (30 °C) cold plantar apparatus for a 30 min habituation period prior to testing (Fig. 3a). Consistent with our prior results, when the mice were exposed to RT for 30 min, we observed a difference in withdrawal latencies between vehicle and QX-314-injected animals (Fig. 3b, *n* = 6–12). However, when the mice were placed on a 30 °C surface for the duration of the experimental protocol (60 min total), no differences in cold sensitivity were observed (*n* = 8), results indicating that temperature was mediating the effects of QX-314 alone.

We then determined the range and efficacies of temperatures in QX-314 block of cold sensitivity. Mice were injected with QX-314 or vehicle and then placed for 30 min on the cold plate set to 10 or 20 °C, then transferred to the heated (30 °C) cold plantar apparatus and lift latencies were recorded every hour starting at 1 h postinjection. At 10 °C, a robust stimulus temperature considered noxious^36^, we observed essentially complete block of cold responses in mice injected with QX-314 up to 3 h postinjection (Fig. 3c). Indeed, at 1 h postinjection, eight of the nine mice tested did not respond prior to the 20 s cutoff time. Cold sensitivity returned to pre-injection levels by 4 h, a time course similar to that observed with QX-314/WS-12 injections performed at RT (Fig. 2a). To determine the TRPM8-dependence of cold-induced uptake of QX-314, we repeated the TRPM8 antagonist strategy described above (Fig. 2 and Supplementary Figure 4a) finding that pre-inhibition of TRPM8 with PBMC blocked cooling (10 °C) mediated effects of QX-314 (Fig. 3d and Supplementary Figure 4b).

As 10 °C is a noxious cold stimulus, we next tested an innocuous cool temperature of 20 °C, again finding robust and essentially complete block of cold responses at 1 and 2 h postinjection. As with 10 °C, six of the eight mice tested did not respond before the cutoff time at 1 and 2 h postinjection. Cold sensitivity increased slightly by 3 h and was at pre-injection levels by 4 h. Thus, QX-314 block of cold sensation remains robust at even mildly innocuous cool temperatures. The efficacy of cooling-induced, QX-314-mediated cold block at 20 °C suggested that this mild stimulus saturated the effects of QX-314 and cold. Thus, we next asked if there was a threshold temperature, repeating the above experiments at 23 and 25 °C. Remarkably, we observed that even these mild temperatures led to modest reductions in cold responsiveness at 1 h after the injection (Fig. 3c, *n* = 8 for both temperatures). These effects were transient as there were no significant differences at 2 h for either temperature (*p* > 0.05). Finally, to gauge the temperature dependence of QX-314-mediated alterations in cold sensitivity, we fitted the latency data at 1 h postinjection for each test temperature with a Boltzmann distribution, finding that 23.1 ± 0.3 °C produced a 50% effective block of cold responses (Supplementary Figure 5). Thus, these data indicate that simple cooling of the QX-314-injection site is sufficient to block nerve conduction of cold-sensitive afferents in a TRPM8-dependent manner.

### Inflammatory and neuropathic cold allodynia and hyperalgesia are alleviated by targeted neuronal silencing

**Fig. 4:**
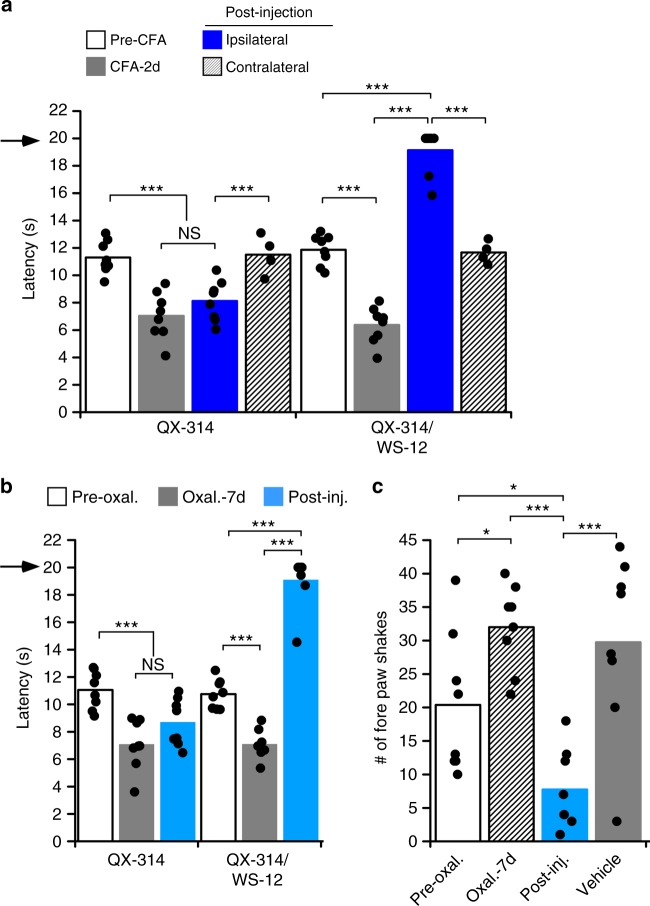
Cold allodynia and hyperalgesia inhibited by targeted silencing with a TRPM8 agonist. **a** Decreased cold-evoked hind paw withdrawal latencies 2 days after 20 µl injection of the inflammatory agent, CFA, were significantly increased after injection with QX-314/WS-12, but not QX-314 (***p < 0.001 vs. pre-CFA, post-CFA, and contralateral paw, ^NS^*p* > 0.05). Arrow denotes the cutoff time for the experiment. **b** Mice that developed cold allodynia 7 days after systemic administration of oxaliplatin (3 mg kg^-1^; ***p < 0.001 pre-oxal. vs. 7 d post-oxal, *n* = 8) showed longer latencies after QX-314/WS-12, but not QX-314 alone (***p < 0.001 post-oxal. vs. post-inj. QX-314/WS-12, ^NS^*p* > 0.05). Arrow denotes the cutoff time for the experiment. **c** Oxaliplatin induced a significant increase in cold hyperalgesia, as measured by cold plate (0 °C) behaviors 7 days post-induction (*p < 0.05 pre- vs. post-oxal, *n* = 8). This cold hyperalgesia was significantly reduced after injection of QX-314/WS-12, but not with vehicle (***p < 0.001 oxal. -7 d vs. post-inj. and post-inj. vs. vehicle injection). Moreover, number of fore paw shakes post-QX-314/WS-12 injection was also significantly different than pre-oxaliplatin (*p < 0.05)

Since acute cold-induced behaviors were successfully blocked after injection of QX-314 and WS-12, we asked if this strategy can mitigate inflammatory and neuropathic cold pain, both allodynia and hyperalgesia, in various mouse pain models. For these experiments mice were maintained at 30 °C for the duration of the experiment to prevent cooling-induced QX-314 uptake. First, we induced inflammatory cold allodynia, measured via the cold plantar assay, with a unilateral injection of complete Freund’s adjuvant (CFA), which leads to an increase (reduced latency) in cold sensitivity by 2 days postinjection (Fig. 4a, *n* = 7–8). However, injection of QX-314/WS-12 into the inflamed hind paw reduced cold sensitivity to a radiant cold stimulus. Indeed, even in the context of cold pain, QX-314/WS-12 was able to completely block cold responses as six of the eight mice did not respond prior to the cutoff time. These latencies were different than those measured pre-CFA and in the contralateral hind paw. Moreover, responses to mechanical (Supplementary Figure 6a) and radiant heat (Supplementary Figure 6b) stimuli remained unaltered postinjection of either QX-314/WS-12 or QX-314 (*p* > 0.05), demonstrating the specificity for cold.

Next we examined a model of CIPN in which cold allodynia and hyperalgesia are prevalent^27,33,37,38^. Wild-type mice received a single intraperitoneal (i.p.) injection of oxaliplatin (3 mg kg^-1^) and then assayed for cold allodynia 7 days postinjection^27,33^. As with inflammation, the robust cold allodynia observed in this pain model (Fig. 4b, *n* = 8) was blocked after QX-314/WS-12 injection and five of the eight mice did not respond prior to the cutoff time, indicating almost complete block of cold sensation.

Finally, we asked if QX-314/WS-12 injections could also ameliorate cold hyperalgesic responses using the fore paw cold plate assay in the oxaliplatin model. As with the cold plantar assay, oxaliplatin treatment led to an increase in cold plate (0 °C) behaviors measured 7 days after the injection (Fig. 4c, *n* = 8). This form of cold hyperalgesia was reduced after injection of WS-12 and QX-314 and the number of behaviors post-QX-314/WS-12 injection were also different than pre-oxaliplatin behaviors, demonstrating that WS-12/QX-314 block of cold remains even under conditions of heightened cold sensitivity. Taken together, these data show that cold pain associated with both allodynia and hyperalgesia can be selectively attenuated by local treatment with TRPM8 agonists and QX-314.

### Cooling induces QX-314-mediated block of cold allodynia

**Fig. 5:**
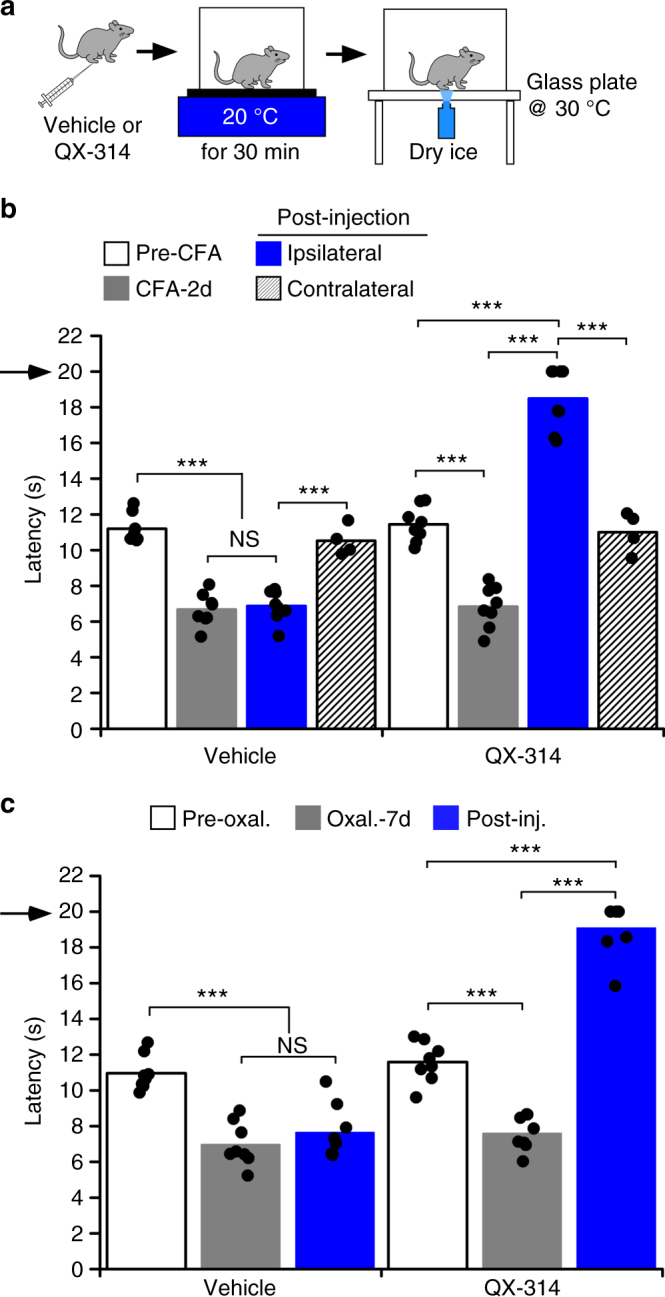
Cooling induces QX-314-mediated block of cold sensitivity after injury. **a** Experimental paradigm for **b**, **c**. After induction of inflammation or CIPN, mice were injected with either vehicle or QX-314 and placed on a surface set at 20 °C for 30 min and then transferred to the cold plantar apparatus in which the glass plate is warmed to 30 °C. **b** Intraplantar injection of QX-314 followed by mild cooling at 20 °C produced an almost complete block of cold responses with inflammation (***p < 0.001 vs. pre-CFA, 2d post-CFA, and the contralateral paw), while vehicle-injected mice were not significantly different from post-CFA (^NS^*p* > 0.05). **c** Exposing the QX-314 injected site to 20 °C for 30 min resulted in a robust decrease in cold sensitivity in mice that developed cold allodynia 7 days after intraperitoneal injection of oxaliplatin (***p < 0.001 post-oxal. vs. pre-oxal. or with QX-314 injection), while the vehicle-injected site showed no differences (^NS^*p* > 0.05 post-oxal. vs. vehicle injection). Arrows denote the cutoff time for the experiments

Next we sought to determine if cold allodynia could be attenuated without necessitating robust activation of cold afferents via TRPM8 agonist activation. Such an approach would be advantageous clinically as it limits the agents to be injected, decreases potential side effects associated with chemical agonists, and allows for a more easily titrated stimulus at the pain site. To this end, after the induction of either CFA-induced inflammation or CIPN, mice were injected with either vehicle or QX-314 then placed on the cold plate set to 20 °C for 30 min, followed by thermal testing on the cold plantar apparatus (Fig. 5a). We chose 20 °C instead of the more robust temperature of 10 °C to limit exposure to this noxious stimulus in these sensitized animals, and as our prior results show that 20 °C was as effective as 10 °C in blocking acute cold responses (Fig. 3c). Remarkably, and results very similar to co-injection of QX-314 with WS-12, modest cooling (20 °C) led to an almost complete block of cold responses (four of eight mice failed to respond before the cutoff time) with latencies different than those prior to the CFA injection, 2 days after CFA injection, and the contralateral hind paw after QX-314 injections (Fig. 5b, *n* = 8). These effects were not observed in vehicle-injected mice subjected to the same experimental conditions. We observed similar results in the CIPN model, again observing almost complete block of cold sensitivity (five of eight mice did not respond before cutoff) and amelioration of the cold allodynic phenotype (Fig. 5c). No effects were observed with vehicle injections, demonstrating that mild cooling with QX-314 abrogates cold sensitivity in both an inflammatory and neuropathic pain setting.

### QX-314-mediated block of cold pain without loss of cold acuity

**Fig. 6:**
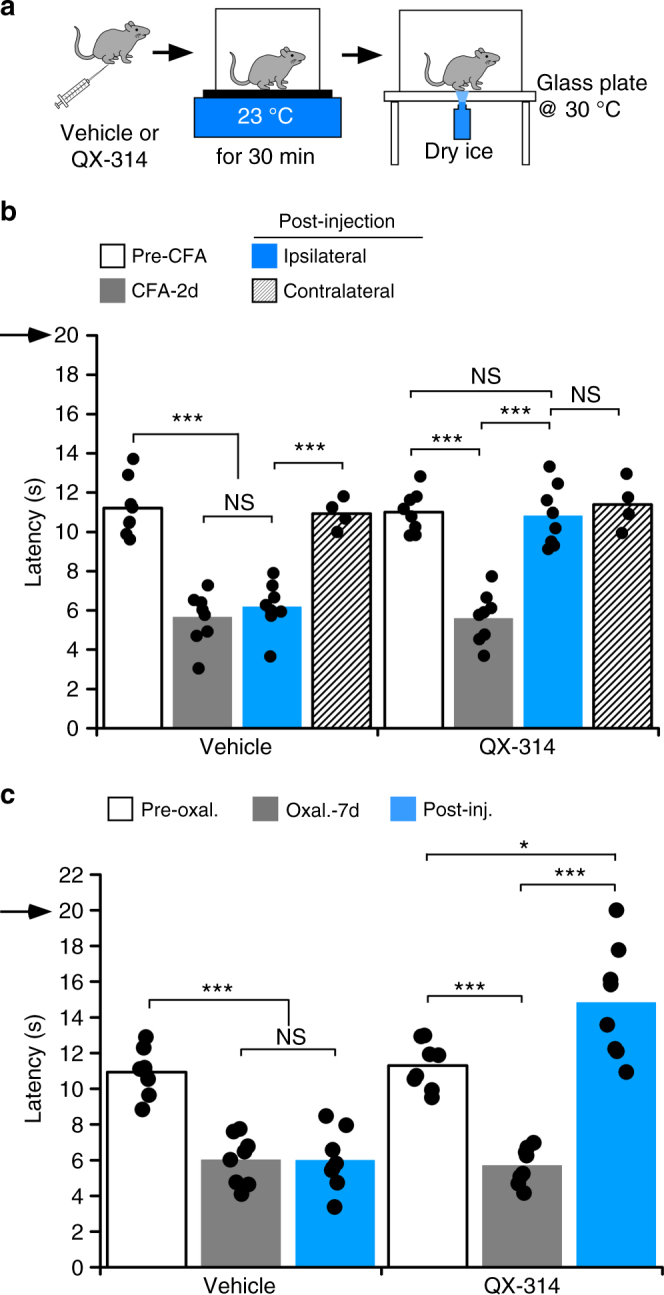
QX-314 mediated block of cold pain to pre-injury states. **a** Experimental paradigm for **b**, **c**. **b** Mild cooling of the QX-314 injected site to 23 °C allows inhibition of cold allodynia during CFA-induced inflammation (***p < 0.001 ipsilateral post-inj. vs. pre-inj., *n* = 8) without diminishing normal cold acuity, bringing withdrawal latencies back to pre-CFA levels or to the levels of the contralateral paw (^NS^*p* > 0.05). Vehicle group with the same treatment developed cold sensitivity after CFA injection (***p < 0.001 vs. pre-CFA or contralateral paw, *n* = 8), but maintained the level of sensitivity to pre-vehicle injection (^NS^*p* > 0.05). **c** Oxaliplatin-induced cold allodynia was abolished in hind paws cooled with 23 °C after QX-314 injection (***p < 0.001 oxal. -7 d vs. post-inj., *n* = 9), however, cold sensitivity did not return to the uninjured state in the ipsilateral paws (*p < 0.05)

While stimulation with either WS-12 or 20 °C leads to amelioration of the cold pain phenotypes observed above, the remarkable robustness of block prevented normal cold perception, an important protective mechanism that is lost under these conditions. As we find that moderately cool temperatures (Fig. 3c) promote QX-314-mediated block of cold sensation, but to a milder extent, we reasoned that these stimuli could lead to block of cold pain but retain relatively normal cold sensation. To this end, we repeated the experiments described above, but with 23 °C as the cooling stimulus (Fig. 6a), finding that cold allodynia associated with inflammation was still blocked with injection of QX-314 (Fig. 6b, *n* = 8). However, cold sensation was largely maintained under these conditions, with the mean latency to lift similar to pre-CFA levels and to that observed for the contralateral control hind paw. Animals injected with vehicle alone showed robust sensitization after the 23 °C stimulus (*n* = 8) and there was no difference in latencies compared to pre-vehicle injection. Moreover, the differences in latencies after QX-314 injection in animals exposed to 20 vs. 23 °C were also statistically significant (*p* < 0.001). These results demonstrate that the stronger cold stimulus blocks all cold sensation, whereas the milder temperature blocks pain without altering the ability to detect cold.

We also examined this stimulus paradigm in mice treated with oxaliplatin, finding that QX-314 and 23 °C also reduced cold allodynia (Fig. 6c, *n* = 9). However, unlike inflammation, cold sensitivity was also reduced compared to that observed prior to oxaliplatin treatment, yet to a lesser extent than when the mice were exposed to 20 °C (Supplementary Figure 6b). We extended these observations to another neuropathic pain model, chronic constriction injury of the sciatic nerve (CCI)^27,39^, finding that similar to the CFA and CIPN models, treatment of these mice with QX-314/WS-12 leads to block of cold and cold pain responsiveness (Supplementary Figure 7, *n* = 4). In addition, cooling of the hind paw with a modest cool stimulus (RT, 22.4 °C) after QX-314 injection blocked cold allodynia and returned lift latencies to that observed prior to CCI, again showing block of pain with retained cold sensation. Finally, we examined both mechanical and heat hypersensitivity in this paradigm, finding that QX-314 and 23 °C treatment had no effect on these modalities (Supplementary Figure 8). These results show that the efficacy of QX-314 mediated block of cold can be titrated by the cooling stimulus used, indicating that cold pain can be attenuated without the complete loss of basal cold sensitivity.

## Discussion

Here we show for the first time that activation of cold-sensitive afferents with TRPM8 agonists or cold in the presence of QX-314 leads to a block of cold sensation in vivo that is dependent on TRPM8 channel function. TRPM8 agonist stimulation with QX-314 has been attempted previously, with reports suggesting that large molecules cannot permeate this TRP channel isoform. For example, an assay for large molecule permeation is the uptake of large dyes in vitro^3^, an approach used by Chen et al.^5^ who observed no uptake when stimulating TRPM8-expressing cells with 100 µM menthol, a concentration near to the EC_50_ that produces submaximal channel activation^5,22^. However, we found that higher concentrations of menthol and the potent and selective TRPM8 agonist WS-12 promote robust uptake of large molecules, results suggesting that the prior study did not sufficiently stimulate TRPM8^31^. Additionally, Nakagawa et al.^30^ concluded that QX-314 did not permeate TRPM8 channels in vivo, a curious conclusion as they only assayed heat sensitivity but not cold behaviors after intradermal injection of QX-314 and menthol. Our current findings clearly show that stimulation with TRPM8 agonists leads to a robust block of cold sensation in vivo, adding this channel to the molecular permeation pathways that can be manipulated for large-cation uptake.

Our results with TRPM8 agonists are consistent with several prior studies that have used TRPV1 activation to selectively block pain behaviors^7,10,11,40^. However, our striking finding that temperature itself can lead to QX-314-dependent block of cold sensation is distinct from TRPV1 as, to the best of our knowledge, heat has not been validated as a stimulus leading to QX-314-block of heat sensitivity. TRPM8 channels are distinct from TRPV1 in that the temperature activation range in vitro spans both innocuous and noxious cold temperatures^22,41^. Moreover, recordings of cold-sensitive afferents both in vivo and in situ show tonic firing of these nerve fibers at ambient temperatures, activity that is dependent on the expression of TRPM8 channels^23,42^. Thus, when QX-314 is injected with the skin exposed to temperatures in the ambient range (22–24 °C), TRPM8 channels are likely active and can facilitate the permeation of QX-314, a posit supported by our data. Heat likely can serve as a stimulus for entry via TRPV1 channels, but such an approach is not attractive as noxious, tissue damaging stimuli would be required. Finally, our findings that the TRPM8 antagonist PBMC blocks cold-induced effects of QX-314 at the noxious cold temperature of 10 °C demonstrates that these extreme temperatures are activating only TRPM8-expressing sensory afferents, further supporting a role for TRPM8 neurons in noxious cold behaviors.

Additionally, our results show selectivity for cold with no alterations in other somatosensory modalities such as heat or force. These results are consistent with the phenotypes of mice lacking either functional TRPM8 channels or intact TRPM8^+^ afferent neurons as these animals have deficits only in cold sensitivity^23,25,27,43^. The ability of cold to induce to QX-314 nerve block, particularly innocuous cool (20 °C) is clinically advantageous over agonists such as capsaicin or WS-12 as these agents instantly activate their respective channels, causing irritation and pain^26,44^. Innocuous temperatures would be perceived as pleasant and be more amenable to long lasting stimulation for more prolonged uptake of the nerve blocker in a clinical setting.

The most intriguing aspect of our results is the select ability to inhibit cold pain associated with inflammation or various forms of painful neuropathy. The temperature at which cold pain is perceived is rather vague, but generally considered to occur at 15 °C and below^45,46^. Cold hypersensitivity occurs in both inflammatory and neuropathic pain conditions such as carpal tunnel syndrome with patients presenting over a 5 °C shift in cold pain thresholds (toward warmer temperatures), a change on par with the lowered heat and mechanical pain thresholds observed in many patients^14,15^. Similarly, the frequency in which patients report cold or mechanical allodynia is nearly identical at 30% and 26%, respectively, in those affected with CRPS^16^. Patients with serious hand injuries or lateral epicondylalgia (LE; arm pain) present with a pathological cold intolerance and lowered cold pain thresholds, respectively, with the occurrence of cold pain as prevalent as mechanical pain^17,18^. Finally, in CIPN a distal cold allodynia is a dose limiting side effect that hinders treatments^13^. Thus, chronic cold pain is as clinically widespread a condition as other pain modalities, yet there are few effective treatments targeting cold pain.

Our findings that QX-314 injection, coupled with either co-injection of WS-12 or stimulation with 20 °C leads to an almost complete block of cold sensation regionally indicates this as a potential treatment method to alleviate localized cold pain. Even more intriguing are the results observed with a very mild stimulus of 23 °C, essentially RT, and the reduction of cold pain without diminished cold acuity. Such an approach is tremendously useful clinically as it is advantageous for the patient to be able to obtain relief from the pain and still be able to perceive the ever-changing environment. Our results are the first to demonstrate that such an outcome may be obtainable via cooling-induced uptake in TRPM8^+^ nerve fibers in the treatment of inflammatory and neuropathic pain.

## Methods

### Reagents

Menthol, QX-314, and oxaliplatin were purchased from Sigma-Aldrich and WS-12 was purchased from Tocris Bioscience. PBMC was purchased from Focus Biochemicals.

### Animals

All wild-type and TRPM8-knockout mice (B57BL/6 background) were purchased from Jackson Laboratories and maintained at University of Southern California animal facility. Mice used in this study were adults between ages of 9- and 12-week-old with equal number of males and females per group. All procedures and tests were approved by the University of Southern California Institutional Animal Care and Use Committee and conducted in accordance with the recommendations of the International Association for the Study of Pain and the NIH Guide for the Care and Use of Laboratory Animals.

### Animal models of pain

To induce inflammation, mice were lightly anesthetized with isoflurane and 20 µL of CFA was administered via unilateral intraplantar injection. Behavioral tests were performed 2 days after injection. To produce CIPN, a single i.p. injection of oxaliplatin (3 mg kg^-1^) was administered^27,33^. Mice were tested for behavioral assays on day 7 post-oxaliplatin injection. Similarly, for the CCI of the sciatic nerve animals were anesthetized with 5% isoflurane for induction, then 3% isoflurane for maintenance^27,33^. The surface of the right hind leg was shaved and sterilized. A two centimeter incision was made in the skin and the muscles overlaying the sciatic nerve gently retracted. Three loose ligatures of 6-0 chromic gut were placed around the nerve proximal to the trifurcation site. The muscles were replaced and the wound closed with VetBond tissue adhesive (3M) and swabbed with topical antibiotic. Animals were monitored for infection and proper wound healing and were tested on day seven or later post surgery.

### Behavioral assays

Vehicle used throughout this study was 10% DMSO in ethanol, with an exception in Supplementary Figure 1, 0.9% saline was used as vehicle. Intraplantar injections of QX-314 into the hind and fore paws contained 0.2 mg of QX-314 in 20 µl (29.1 mM, 1%) and 0.2 mg of QX-314 in 10 µl (58.1 mM, 2%), respectively. Intraplantar injections with WS-12 consisted of 3.75 mM WS-12 in 20 µl for the hind paws and 7.5 mM WS-12 in 10 µl for the fore paws. Intraplantar injections with menthol consisted of 2.56 mM menthol in 20 µl.

### Cold plantar assay

To measure cold sensitivity of the hind paws, the cold plantar assay was performed as previously described^27,33^. Briefly, mice were allowed to acclimate in Plexiglas chambers for 2 h prior to cold plantar testing performed at RT or 30 min prior to cold plantar testing performed at a glass surface of 30 °C. A dry ice pellet was then applied to the hind paw of the mice through a glass at a thickness of 6 mm. Hind paw withdrawal latencies were recorded for a total of three trials per paw for each time point.

### Cold plate assay

To assess responses to noxious cold, mice were placed in a Plexiglas chamber on a hot/cold plate apparatus (IITC) with plate temperature set at 0 °C and video recorded for 3 min for a later quantification^27^. Total number of fore paw flinches were quantified by observers blind to the solution injected.

### Electronic Von Frey

To measure sensitivity to mechanical stimulation, electronic Von Frey assay was performed as previously described^27^. Briefly, mice were placed in Plexiglas chamber on an elevated mesh platform and acclimated for 20 min. The force at which the mouse withdrew the paw was measured upon stimulation with a semiflexible tip raised to the plantar surface of the hind paw by electronic Von Frey apparatus (IITC). Hind paw withdrawal thresholds were averaged from three trials per paw.

### Hargreaves

To test heat sensitivity, the Hargreaves assay was performed^27^. Mice were allowed to acclimate in Plexiglas chamber on a glass plate heated to 32 °C for 30 min. Hind paw withdrawal latencies were measured. The basal paw withdrawal latency was adjusted to 9–12 s, with a cutoff time of 20 s to avoid tissue damage. The paw withdrawal latency was averaged from three trials per paw.

### Statistical analysis

For the initial experiments testing effects of the different treatments with the cold plantar assay (Figs. 1–3), the experimenter was blinded to the substances injected when performing the behavioral assays, with all subsequent experiments performed unblinded. For the cold plate assays, the experiments were quantified by an individual blinded to the experimental conditions. All results from behavioral assays were expressed as mean ± SEMs for each group, as well as experimental numbers, as indicated in the text and figure legends. The sample size in each experiment was based on our previous studies on similar experiments^27^. Statistical analysis was completed with Origin or Excel. Behavioral data were analyzed using either a paired or two sample student’s *t*-test as appropriate with equal variance not assumed (Welch Correction). For temporal assays data were analyzed by two‐way repeated measures of ANOVA, followed by post hoc Bonferroni analysis. The criterion for statistical significance was *p* < 0.05.

### Data availability

All data generated or analyzed during this study are included in this published article (and its supplementary information files) or are available upon reasonable request.

## Electronic supplementary material

Supplementary Information
