# Supplementary material for: Selective cold pain inhibition by targeted block of TRPM8-expressing neurons with quaternary lidocaine derivative QX-314
Source: Commun Biol. 2018 May 31;1:53. doi: 10.1038/s42003-018-0062-2 (PMC6123689; doi:10.1038/s42003-018-0062-2)
Supplement: Supplementary file 1 — Supplementary Information [file 42003_2018_62_MOESM1_ESM.pdf]

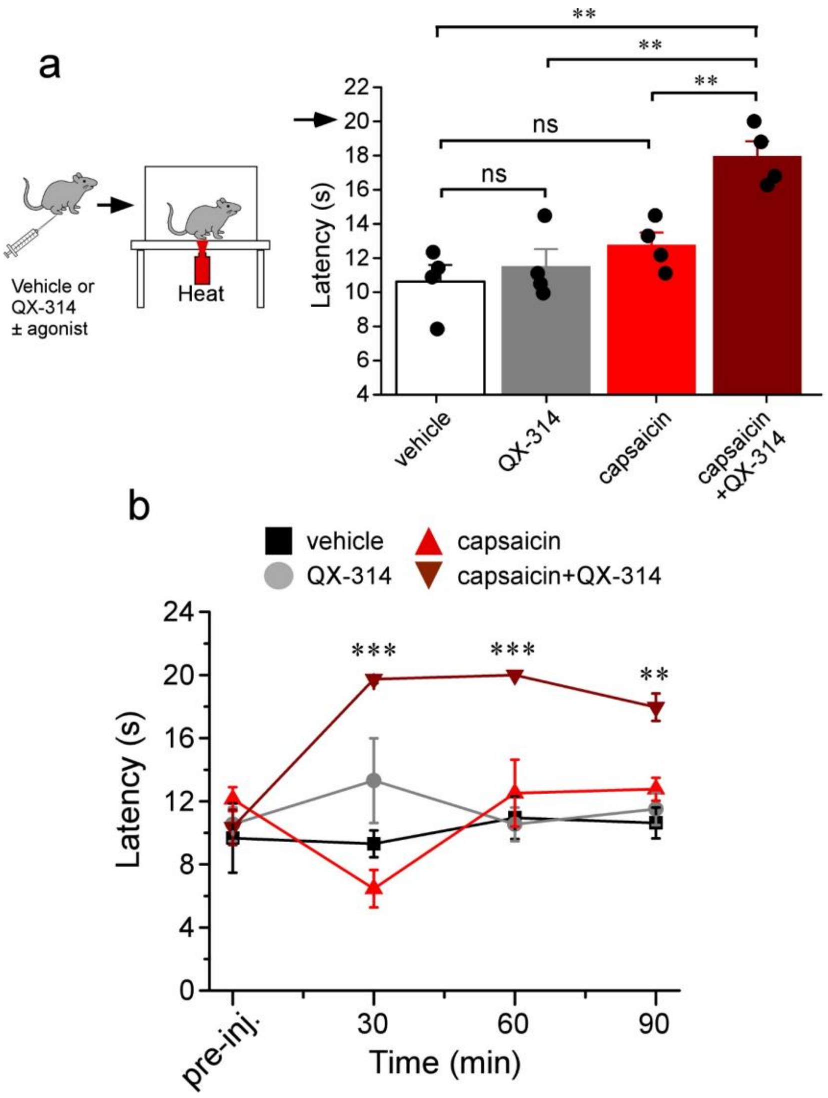

Supplementary Figure 1. Loss of heat sensation after capsaicin and QX-314 intraplantar injection. (**a**) Time course of hind paw lift latencies to a radiant heat stimulus after intraplantar injections of vehicle, 1μg capsaicin, 1% QX-314-314, or capsaicin/QX-314 shows that the effects of capsaicin/QX-314 injection were produced as early at 30min post injection (***p<0.001, **p<0.01 vs pre-inj). (**b**) Summary shows a significantly longer lift latency to a radiant heat stimulus compared to vehicle, QX-314 alone, or capsaicin alone measure 90min post-injection (**p<0.01, n=4).

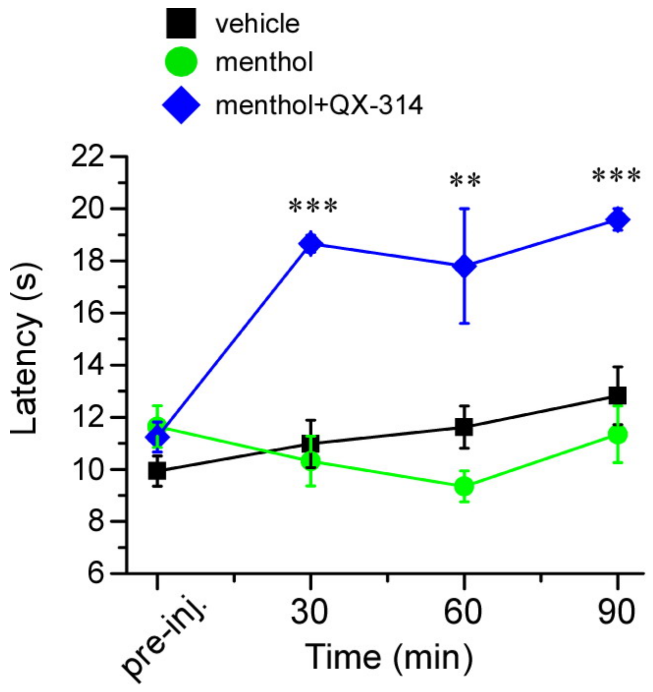

Supplementary Figure 2. Loss of cold sensation after menthol and QX-314 intraplantar injection. Time course of the data in Fig. 1a shows that the effects of menthol/QX-314 injection were produced as early at 30min post injection (***p<0.001, **p<0.01 vs pre-inj).

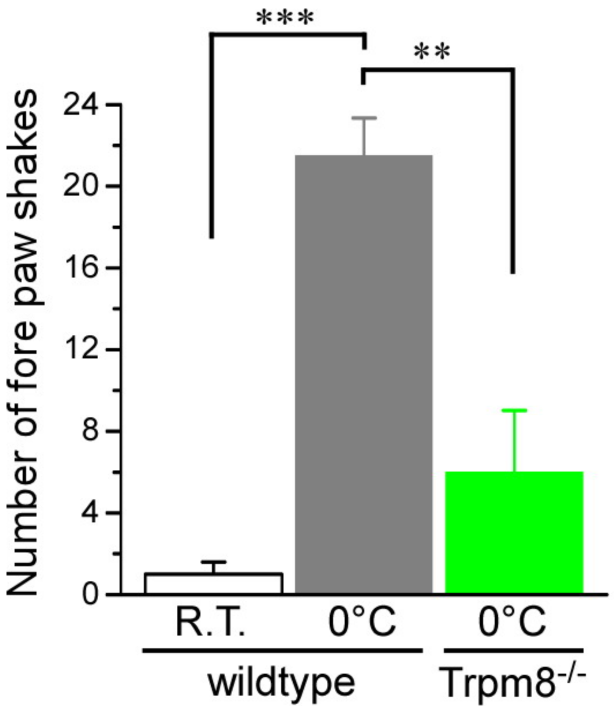

Supplementary Figure 3. Fore paw nocifensive behaviors are TRPM8-dependent. Comparison of the number of fore paw shakes observed over a 3min. recording period in wildtype mice placed on a plate set to either room temperature (R.T.) or 0°C (***p<0.001 wildtype R.T. vs 0°C, n=4-8). Trpm8$^{-/-}$ mice show significantly less fore paw shakes at 0°C compared to wildtype mice (**p<0.01).

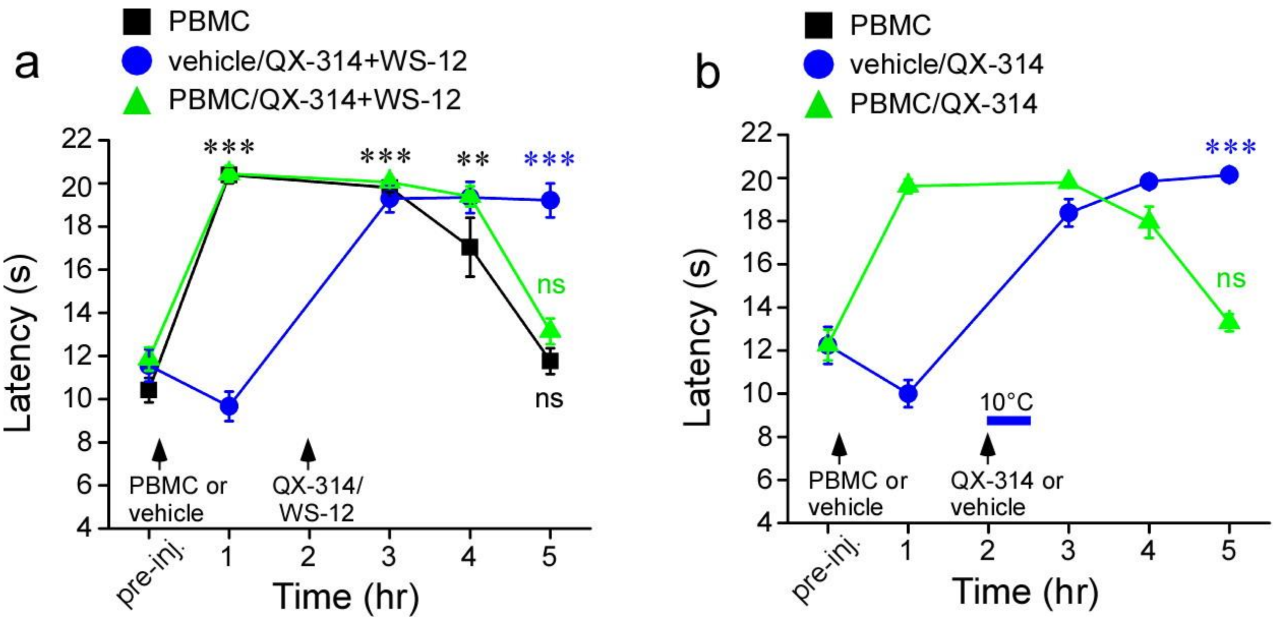

Supplementary Figure 4. TRPM8 antagonism blocks QX-314-mediated inhibition of cold sensitivity. (**a**) Wildtype mice injected with 100µg PBMC alone (in 20µl, black boxes) are cold-insensitive for up to 4hrs post injection (***p<0.001, **p<0.01 vs pre-inj, n=4) with latencies to lift returning to pre-injection values by 5hrs (^{ns}p>0.05). When a PBMC or vehicle pre-injection was performed 2hrs before injection of QX-314/WS-12 (as in Fig. 2a), cold sensitivity was blocked 3hrs later in animals pre-injected with vehicle (***p<0.001 vs pre-injection) but not when injected with PBMC (^{ns}p>0.05 vs pre-injection, n=5-6). (**b**) Experimental protocol similar to (**a**) except mice were stimulated with 10°C (blue bar) 2hrs post PBMC or vehicle injection (^{ns}p>0.05, ***p<0.001 vs pre-injection, n=6).

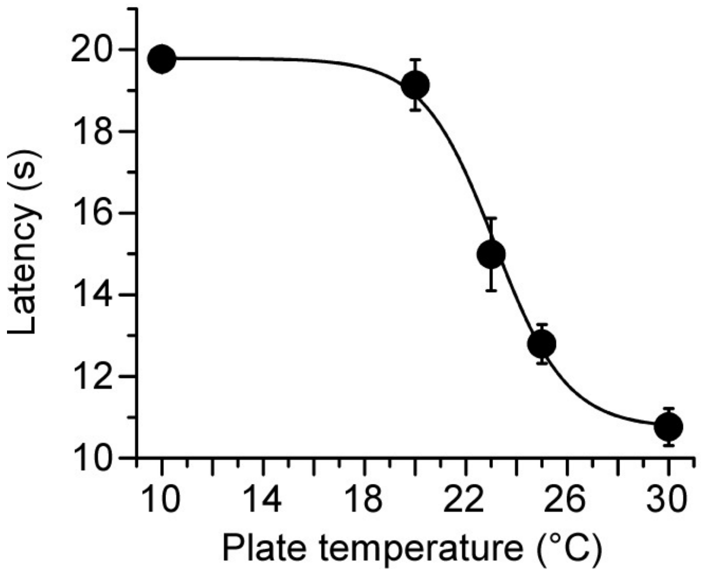

Supplementary Figure 5. Temperature range of QX-314 mediated inhibition of cold sensation. Data from Fig. 3c at 1hr post injection was used to derive a Boltzmann distribution of the lift latency versus temperature.

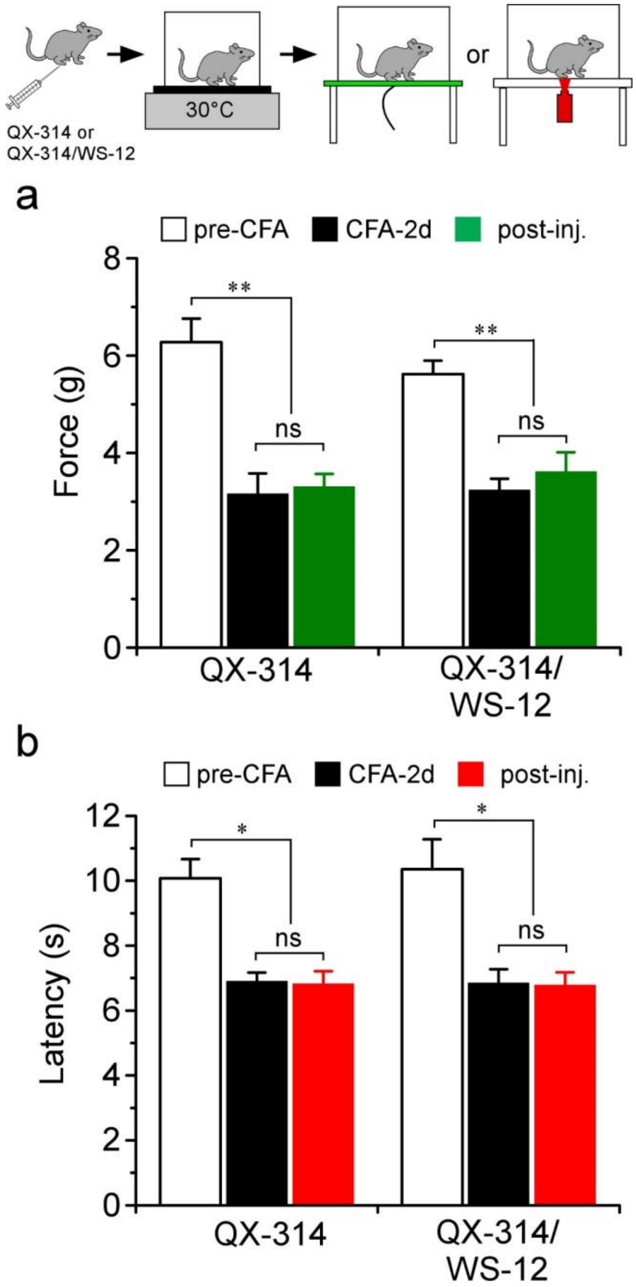

Supplementary Figure 6. Mechanical and heat sensation are not altered with injection of QX-314 and WS-12. Mice in which inflammation was induced via CFA injection showed increased sensitivity a mechanosensory (**a**) or heat stimulus (**b**) assayed 3 days post-CFA (**p<0.01,*p<0.05, n=4). Injection of QX-314 or QX-314/WS-12 as described in Fig. 5 produced no change in either mechanical or heat sensation (^{ns}p>0.05).

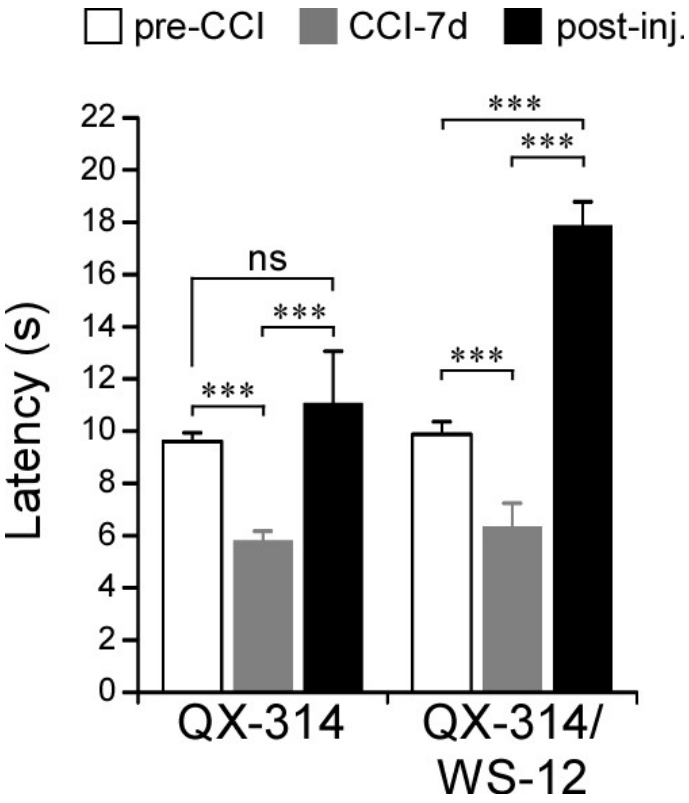

Supplementary Figure 7. WS-12 and cooling induces QX-314-mediated block of cold sensitivity after nerve injury. The CCI of the sciatic surgery was performed on wildtype mice, leading to cold allodynia by 7d post-surgery (***$p<0.001$, $n=4$). Injection of QX-314 alone or QX-314/WS-12 led to a significant increase in lift latencies compared to pre-injection and before the CCI surgery for mice treated with QX-314 and WS-12 (***$p<0.001$). Mice injected with QX-314 alone and placed on a cool plate prior to testing showed lift latencies similar to pre-CCI ($^{ns}p>0.05$).

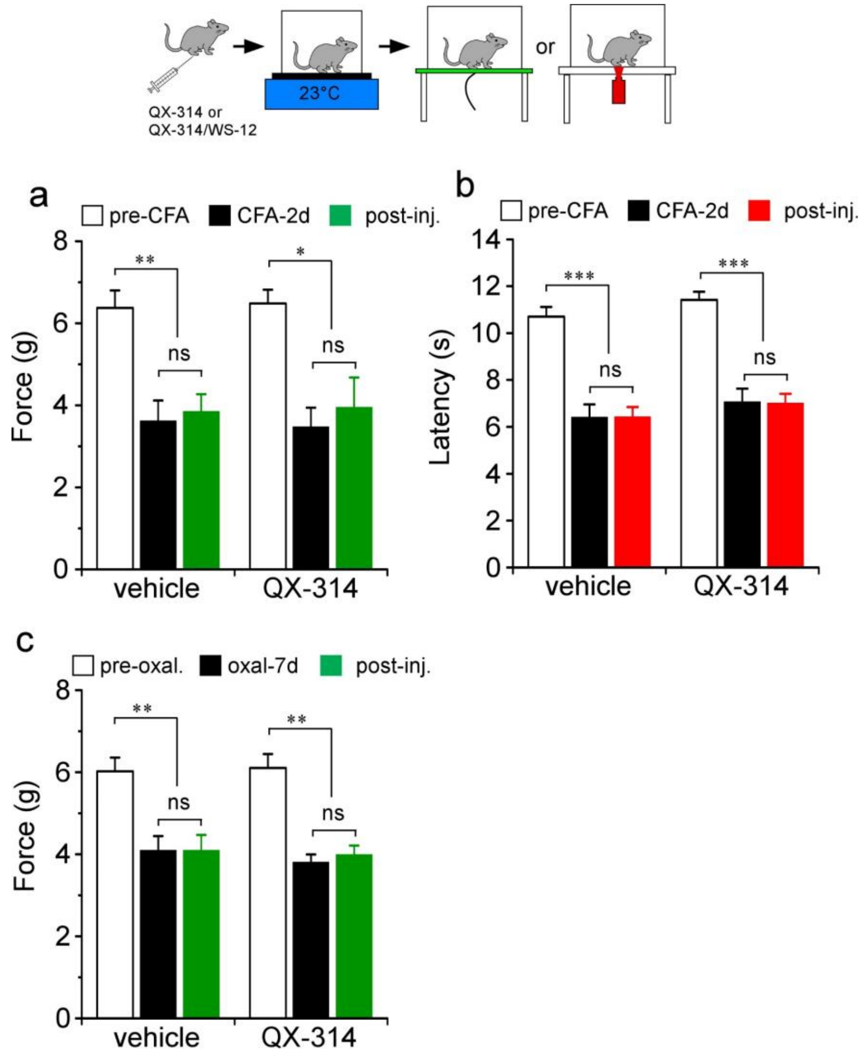

Supplementary Figure 8. Cooling after injection of QX-314 does not alter inflammatory or neuropathic mechanical and heat sensitization. Mice in which inflammation was induced via CFA injection showed increased sensitivity a mechanosensory (**a**) or heat stimulus (**b**) assayed 3 days post-CFA (**$p<0.01$,*$p<0.05$, n=4). Injection of vehicle or QX-314, along with cooling at 23°C as described in Fig. 6 produced no change in either mechanical or heat sensation ($^{ns}p>0.05$). (**c**) Similarly, mice injected with oxaliplatin showed robust mechanical sensitization 7d post injection (**$p<0.01$, n=8), with no change after either vehicle or QX-314 injection with the 23°C stimulus ($^{ns}p>0.05$).
